# Association between CYP1A1 Ile462Val Variation and Acute Leukemia Risk: Meta-Analyses Including 2164 Cases and 4160 Controls

**DOI:** 10.1371/journal.pone.0046974

**Published:** 2012-10-04

**Authors:** Wenlei Zhuo, Liang Zhang, Bo Zhu, Zhiqun Qiu, Zhengtang Chen

**Affiliations:** 1 Institute of Cancer, Xinqiao Hospital, Third Military Medical University, Chongqing, China; 2 Department of Environmental Hygiene, College of Preventive Medicine, Third Military Medical University, Chongqing, China; University of Navarra, Spain

## Abstract

**Background:**

Previously, CYP1A1 Ile462Val polymorphism has been indicated to be a risk factor for several malignancies. Increasing reports have focused on the association of CYP1A1 Ile462Val polymorphisms with susceptibility to acute leukemia and have generated controversial results. The goal of the present study was to derive a more precise estimation of the relationship.

**Methods:**

Relevant literature has been rigorously searched and screened. Eligible studies were identified for the period up to Apr 2012. Meta-analyses evaluating the association of CYP1A1 Ile462Val variation with acute leukemia were carried out. Subgroup analyses on ethnicity, clinical types and source of controls were further performed.

**Results:**

A total of thirteen publications including fourteen case-control studies with 2164 cases and 4160 controls were selected for analysis. The overall data indicated a significant association of CYP1A1 Ile462Val polymorphism with acute leukemia risk (Val/Val vs Ile/Ile OR = 1.49; 95% CI = 1.11–1.98; dominant model: OR = 1.26; 95% CI = 1.05–1.51; recessive model: OR = 1.38; 95% CI = 1.04–1.83). In subgroup analysis on ethnicity, increased risk was shown among mixed ethnicities (Val/Val vs Ile/Ile: OR = 2.36; 95% CI = 1.46–3.82; dominant model: OR = 1.37; 95% CI = 1.01–1.86; recessive model: OR = 2.20; 95% CI = 1.37–3.53) but not Asians or Caucasians. In subgroup analysis on clinical types, increased risk was observed in the acute lymphocytic leukemia (ALL) subgroup (Val/Val vs Ile/Ile: OR = 2.06; 95% CI = 1.42–3.01; recessive model: OR = 1.91; 95% CI = 1.32–2.76) but not in the acute myeloid leukemia (AML) subgroup.

**Conclusion:**

The results of the present study suggest that CYP1A1 Ile462Val polymorphism might be a low-penetrant risk factor for acute leukemia. Subgroup analyses suggest that homozygous Val/Val alleles might modify the susceptibility to ALL.

## Introduction

Acute leukemia, a malignant tumor of the hematopoietic system, is characterized by a rapid increase in the numbers of immature blood cells. The disease can be subdivided into two major groups according to the cell affected as acute lymphoblastic leukemia (ALL) and acute myeloid leukemia (AML), respectively. ALL is the most common type of leukemia in young children while AML occurs more commonly in adults than in children [**1**], [**2**]. The mechanisms for acute leukemia genesis are not fully understood. Previous evidence suggests that radiation, smoking, obesity and exposure to chemical carcinogens are considered as its risk factors [**3**]. Nevertheless, though individuals are exposed to these environmental and lifestyle risk factors, acute leukemia develops only in a small proportion of the exposed people, indicating that the host genetic factors might play an important role in the genesis of leukemia.

Several genetic variations have been evaluated as possible risk factors for leukemia by meta-analyses. Polymorphisms of GSTM1, GSTT1, MTHFR C677T and XRCC1 Arg399Gln have been indicated to increase leukemia risk [**4**], [**5**], [**6**]. However, significant associations of polymorphic MTR A2756G with decreased acute leukemia susceptibility were found [**7**]. Thus, different genetic polymorphisms exert different effects on acute leukemia risk. Nevertheless, only a few gene polymorphisms associated with leukemia susceptibility have been identified. To explore the roles of other genetic polymorphisms on the risk is required.

Previous evidence indicates that carcinogen-metabolizing genes may play critical roles in determining individual susceptibility to malignancies [**8**], [**9**]. Genetic variations in these genes may change the activities of their encoded enzymes, possibly by altering their expression and function. Cytochrome P450 enzymes catalyze Phase I metabolism reaction. Cytochrome P450 1A1 (CYP1A1) is a member of the CYP1 family that participates in the metabolism of xenobiotics and endogenous compounds, particularly polycyclic aromatic hydrocarbons (PAHs) such as benzo[a]pyrene [**10**]. A commonly investigated single nucleotide polymorphism (SNP) in the CYP1A1 gene has been suggested to have a correlation with cancer risk. The SNP leads to a base substitution of isoleucine with valine at codon 462 in exon7 (Ile462Val or CYP1A1*2C polymorphism, rs1048943). Thus, the exon7 restriction site polymorphism results in three genotypes: a predominant homozygous Ile/Ile, the heterozygote Ile/Val and a rare homozygous Val/Val [**11**].

A number of published studies have been conducted on the relationship between CYP1A1 Ile462Val polymorphism and acute leukemia risk. However, the results are inconclusive. The issue of whether the CYP1A1 Ile462Val polymorphism is a risk factor for acute leukemia has not been clearly addressed. Thus, in this study we aimed to derive a more precise estimation of the relationship by performing a quantitative meta-analysis that increases statistical power to reach more convincible results.

## Materials and Methods

### 1 Literature search Strategy

To obtain eligible literature, we carried out a search in the Medline, EMBASE, OVID, Sciencedirect, Google Scholar and Chinese National Knowledge Infrastructure (CNKI) without a language limitation, covering published publications up to Apr 2012, with a combination of the following keywords: *Cytochrome P450 1A1, CYP1A1, Ile462Val, exon7, acute leukemia, hematology, malignancy, neoplasm, cancer, variation* and *polymorphism*. All searched studies were retrieved and the bibliographies were checked for other relevant publications. Review articles and bibliographies of other relevant studies identified were electric/hand searched to find additional eligible studies.

### 2 Inclusion and Exclusion Criteria

The following criteria were used for the literature selection: first, studies should concern the association of CYP1A1 Ile462Val polymorphism with acute leukemia risk; second, studies must be observational studies (Case–control or cohort); third, papers must offer the sample size, odds ratios (ORs) and their 95% confidence intervals (CIs), the genetic distribution or the information that can help infer the needed results. Accordingly, the following criteria for exclusion were also used: first, the design and the definition of the experiments were obviously different from those of the selected articles; second, the source of cases and controls and other essential information were not offered; third, reviews and duplicated publications. After rigorous searching, we reviewed all papers in accordance with the criteria defined above for further analysis.

### 3 Data Extraction

Data were carefully extracted from all eligible publications independently by two of the authors **(Zhuo and Zhang)** according to the inclusion criteria mentioned above. For conflicting evaluations, an agreement was reached following a discussion. If a consensus could not be reached, another author was consulted to resolve the dispute and then a final decision was made by the majority of the votes. The extracted information was entered into a database.

### 4 Statistical Analysis

The odds ratio (OR) of CYP1A1 Ile462Val polymorphisms and acute leukemia risk was estimated for each study. The pooled ORs were performed for a homozygote comparison model (Val/Val versus Ile/Ile), a dominant model (Val/Val+Val/Ile versus Ile/Ile) and a recessive model (Val/Val versus Val/Ile+Ile/Ile), respectively. To detect any possible sample size biases, the OR and its 95% confidence interval (CI) to each study was plotted against the number of participants respectively. *I^2^* value was applied to evaluate heterogeneity between the included studies (*I^2^* = 0–25%, no heterogeneity; *I^2^* = 25–50%, moderate heterogeneity; *I^2^*>50%, large heterogeneity) [**12**] **.** In addition, a Chi-square based Q statistic test was conducted to assess the heterogeneity. If the result of the Q-test was *P*>0.1, ORs were pooled according to the fixed-effect model (Mantel-Haenszel), Otherwise, the random-effect model (DerSimonian and Laird) was used. The significance of the pooled ORs was determined by Z-test. The Hardy-Weinberg equilibrium (HWE) was assessed by Fisher’s exact test. Publication bias was assessed by visual inspection of funnel plots [**13**], in which the standard error of log (OR) of each study was plotted against its log (OR). An asymmetric plot indicates a possible publication bias. The symmetry of the funnel plot was further evaluated by Egger’s linear regression test [**14**]. Statistical analysis was undertaken using the program STATA 11.0 software (Stata Corporation, Texas, USA).

## Results

### 1 Study Characteristics

**Figure 1:**
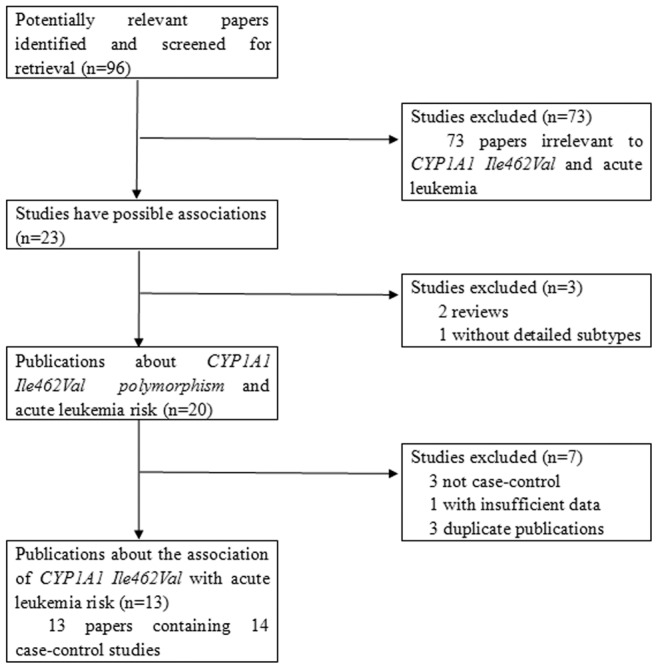
The flow diagram of included/excluded studies.

Relevant publications were retrieved and screened carefully. A total of ninety-six publications were identified, of which seventy-three irrelevant papers were excluded. As shown in **Figure 1**, twenty-three publications were preliminary eligible, of which two review articles [**15**], [**16**] and one publication without detailed subtypes of leukemia [**17**] were then excluded. Next, three publications not being case-control studies [**18**], [**19**], [**20**] and one article without sufficient data [**21**] were discarded. As a result, sixteen publications were selected for data extraction. However, three duplicate publications [**16**], [**22**], [**23**] which concerned the same research with one selected study [**24**] were further excluded. Moreover, an included article provided two separated groups of data regarding childhood leukemia and adult leukemia, respectively [**25**]. Thus, each group was considered as a separate study for analysis. Lastly, thirteen publications containing fourteen case-control studies were selected [**24**], [**25**], [**26**], [**27**], [**28**], [**29**], [**30**], [**31**], [**32**], [**33**], [**34**], [**35**], [**36**].

Of the selected publications, one publication was written in Chinese [**26**] while the remaining twelve were in English. The relevant information was listed in **Table 1**. According to this table, the first author and the number and characteristics of cases and controls for each study as well as other necessary information were presented. There were three groups of Caucasians [**24**], [**27**], [**34**], five of Asians [**26**], [**29**], [**31**], [**32**], [**36**] and six of mixed ethnicities [**25**], [**28**], [**30**], [**33**], [**35**] in this meta-analysis. As shown in **Table 1**, five groups of AML [**25**], [**27**], [**31**], [**33**], [**36**] and nine of ALL [**24**], [**25**], [**26**], [**28**], [**29**], [**30**], [**32**], [**34**], [**35**] were included in this study. As for age groups, there were eight childhood ALL [**24**], [**25**], [**26**], [**29**], [**30**], [**32**], [**34**], [**35**] and one adult ALL [**28**] groups in this study. All AML studies concerned adult AML.

**Table 1:** Characteristics of studies included in the meta-analysis.

First Author	Publication Year	Number of Cases (male/female)	Number of Controls (male/female)	Subtypes of cases	Type of controls	Median (or mean) age, (range) year (Cases/Controls)	Racial decent	Country
Krajinovic	1999	177 (110/67)	304 (144/160)	177 ALL	Healthy controls (PB)	8(1–21)/NA	Caucasian	Canada
Gao	2003	78 (40/38)	112 (58/54)	78 ALL	Healthy controls (PB)	NA(2–15)/NA(3–15)	Asian	China
D’Alo	2004	193 (107/86)	273(147/126)	193 AML	Healthy controls (PB)	62(19–87)/60(19–90)	Caucasian	Italy
Gallegos-Arreola	2004	136 (57/79)	136 (66/70)	136 ALL	Healthy controls (PB)	37.5(18–78)/41.9(18–72)	Mixed	Mexico
Joseph	2004	118 (77/41)	118 (77/41)	118 ALL	Non-cancer controls (age,- sex-matched; HB)	NA(0–14)/NA(0–14)	Asian	India
Selvin	2004	175 (NA)	175 (NA)	175 ALL	Healthy controls (PB)	NA(0–14)/NA(0–14)	Mixed	USA
Majumdar	2008	110 (70/40)	126 (54/72)	110 AML	Healthy controls (PB)	35(4–81)/30(8–73)	Asian	India
Lee	2009	164 (101/63)	164 (101/63)	106 ALL	Non-cancer controls (age,- gender-matched; HB)	6.96(0–18)/7.09(0–18)	Asian	Korea
Yamaguti	2009	133 (70/63)	133 (70/63)	133 AML	Healthy controls (PB)	47(11–89)/53(25–60)	Mixed	Brazil
Yamaguti	2010	99 (52/47)	99 (52/47)	99 ALL	Healthy controls (PB)	4(NA)/53(NA)	Caucasian	Brazil
Razmkhah (Adult)	2011	144 (77/67)	95 (52/43)	105 AML	Healthy controls (PB)	45.7(NA)/44.8(NA)	Mixed	Iran
Razmkhah (Childhood)	2011	85 (47/38)	94 (53/41)	85 ALL	Healthy controls (PB)	5.6(0–16)/6.2(0–16)	Mixed	Iran
Swinney	2011	258 (137/121)	646 (NA)	258 ALL	Healthy controls (age-, gender-, ethnicity-matched; PB)	6.3(NA)/NA	Mixed	USA
Kim	2012	415 (223/192)	1700 (821/879)	415 AML	Healthy controls (PB)	50.5(15–86)/52.2(20–74)	Asian	Korea

NA: not available; AML, acute myeloid leukemia; ALL, acute lymphocytic leukemia; PB: population-based; HB: hospital-based.

The distributions of CYP1A1 Ile462Val genotypes as well as the genotyping methods of the included studies were presented in **Table 2**. The genetic distributions of the control groups in all studies were consistent with HWE except for one study [**35**].

**Table 2:** Distribution of CYP1A1 Ile462Val genotype among acute leukemia cases and controls included in the meta-analysis.

First Author	Year	Genotyping method	Cases			Controls			HWE (control)	
			Val/Val	Val/Ile	Ile/Ile	Val/Val	Val/Ile	Ile/Ile	Chi-squre	P
Krajinovic	1999	PCR-RFLP	1	11	158	0	24	275	0.523	>0.05
Gao	2003	ASA	10	44	24	11	59	42	2.226	>0.05
D’Alo	2004	PCR-RFLP	0	14	179	0	25	248	0.629	>0.05
Gallegos-Arreola	2004	PCR	22	65	49	8	59	69	1.010	>0.05
Joseph	2004	PCR-RFLP	10	34	74	3	20	95	2.168	>0.05
Selvin	2004	Not determined	5	39	131	4	44	127	0.007	>0.05
Majumdar	2008	PCR-RFLP	1	24	85	3	18	105	3.687	>0.05
Lee	2009	SNaPshot	6	39	60	9	65	85	0.567	>0.05
Yamaguti	2009	PCR-RFLP	4	54	75	2	39	92	0.891	>0.05
Yamaguti	2010	PCR-RFLP	3	36	60	3	29	67	0.004	>0.05
Razmkhah (Adult)	2011	PCR-RFLP	1	38	66	1	18	76	0.003	>0.05
Razmkhah (Childhood)	2011	PCR-RFLP	0	13	72	0	14	80	0.609	>0.05
Swinney	2011	Golden gate assay	14	40	188	18	118	505	10.693	<0.05
Kim	2012	PCR-RFLP	17	173	225	87	654	959	3.311	>0.05

### 2 Test of Heterogeneity

As shown in **Table 3**, evident heterogeneities were observed for the overall data in the dominant model (P = 0.049 for Q-test; *I^2^* = 42.1%), except for the homozygote comparison (P = 0.171 for Q-test; *I^2^* = 27.9%) and the recessive models (P = 0.247 for Q-test; *I^2^* = 20.1%). However, when subgroup analyses on ethnicity, clinical types and source of controls were further conducted, we found diminished heterogeneities in some of the subgroups under the dominant model.

**Table 3:** Main results of the pooled data in the meta-analysis.

	No. (cases/controls)		Val/Val vs Ile/Ile				(Val/Val+Val/Ile) vs Ile/Ile				Val/Val vs (Val/Ile+Ile/Ile)			
			OR (95%CI)	P	P (Q-test)	I^2^	OR (95%CI)	P	P (Q-test)	I^2^	OR (95%CI)	P	P (Q-test)	I^2^
Total	2164/4160		1.49 (1.11–1.98)	0.007	0.171	27.9%	1.26 (1.05–1.51)	0.011	0.049	42.1%	1.38 (1.04–1.83)	0.025	0.247	20.1%
Clinical types														
ALL	1208/1833		2.06 (1.42–3.01)	0.000	0.448	0.0%	1.22 (0.96–1.54)	0.098	0.097	40.5%	1.91 (1.32–2.76)	0.001	0.601	0.0%
AML	956/2327		0.89 (0.55–1.44)	0.633	0.603	0.0%	1.35 (0.97–1.89)	0.076	0.061	55.6%	0.83 (0.51–1.34)	0.439	0.672	0.0%
Age group (ALL)														
Adult ALL		136/136	3.87 (1.59–9.41)	0.003	–	–	1.83 (1.13–2.97)	0.015	–	–	3.09 (1.32–7.21)	0.009	–	–
Childhood ALL		1072/1697	1.76 (1.15–2.69)	0.009	0.638	0.0%	1.15 (0.91–1.45)	0.258	0.179	31.2%	1.68 (1.11–2.53)	0.015	0.701	0.0%
Ethnicity														
Caucasian		462/671	1.60 (0.39–6.55)	0.512	0.400	0.0%	1.01 (0.69–1.48)	0.945	0.418	0.0%	1.47 (0.37–5.95)	0.586	0.362	0.0%
(ALL)		269/398	1.60 (0.39–6.55)	0.512	0.400	0.0%	1.14 (0.72–1.79)	0.571	0.344	0.0%	1.47 (0.37–5.95)	0.586	0.362	0.0%
(AML)		193/273	–	–	–	–	0.78 (0.39–1.53)	0.466	–	–	–	–	–	
Asian		826/2215	1.09 (0.74–1.61)	0.646	0.173	37.2%	1.29 (0.94–1.76)	0.115	0.072	53.6%	1.02 (0.70–1.49)	0.901	0.247	26.2%
(ALL)		301/389	1.73 (0.94–3.21)	0.080	0.221	33.8%	1.40 (0.76–2.59)	0.286	0.029	71.9%	1.57 (0.87–2.83)	0.138	0.327	10.6%
(AML)		525/1826	0.80 (0.47–1.35)	0.403	0.556	0.0%	1.13 (0.92–1.38)	0.256	0.393	0.0%	0.76 (0.45–1.27)	0.298	0.533	0.0%
Mixed		876/1274	2.36 (1.46–3.82)	0.000	0.642	0.0%	1.37 (1.01–1.86)	0.041	0.064	52.0%	2.20 (1.37–3.53)	0.001	0.791	0.0%
(ALL)		638/1046	2.41 (1.45–4.01)	0.001	0.323	11.6%	1.16 (0.85–1.60)	0.348	0.182	38.4%	2.28 (1.38–3.77)	0.001	0.523	0.0%
(AML)		238/228	2.00 (0.47–8.53)	0.348	0.651	0.0%	1.95 (1.31–2.90)	0.001	0.457	0.0%	1.64 (0.39–6.92)	0.502	0.628	0.05
Source of controls														
PB		1941/3883	1.44 (1.05–1.96)	0.022	0.207	25.7%	1.23 (1.04–1.46)	0.015	0.178	27.2%	1.33 (0.98–1.81)	0.064	0.247	21.3%
(ALL)		985/1556	2.14 (1.39–3.29)	0.001	0.600	0.0%	1.17 (0.96–1.43)	0.118	0.424	0.0%	1.96 (1.29–2.97)	0.002	0.661	0.0%
(AML)		956/2327	0.89 (0.55–1.44)	0.633	0.603	0.0%	1.35 (0.97–1.89)	0.076	0.061	55.6%	0.83 (0.51–1.34)	0.439	0.672	0.0%
HB		223/277	1.83 (0.83–4.02)	0.132	0.083	66.7%	1.44 (0.51–4.01)	0.489	0.008	85.9%	1.74 (0.80–3.82)	0.164	0.144	53.1%
(ALL)		223/277	1.83 (0.83–4.02)	0.132	0.083	66.7%	1.44 (0.51–4.01)	0.489	0.008	85.9%	1.74 (0.80–3.82)	0.164	0.144	53.1%
(AML)		–	–	–	–	–	–	–	–	–	–	–	–	–

AML, acute myeloid leukemia; ALL, acute lymphocytic leukemia; PB: population-based; HB: hospital-based.

### 3 Meta-analysis Results

**Figure 2:**
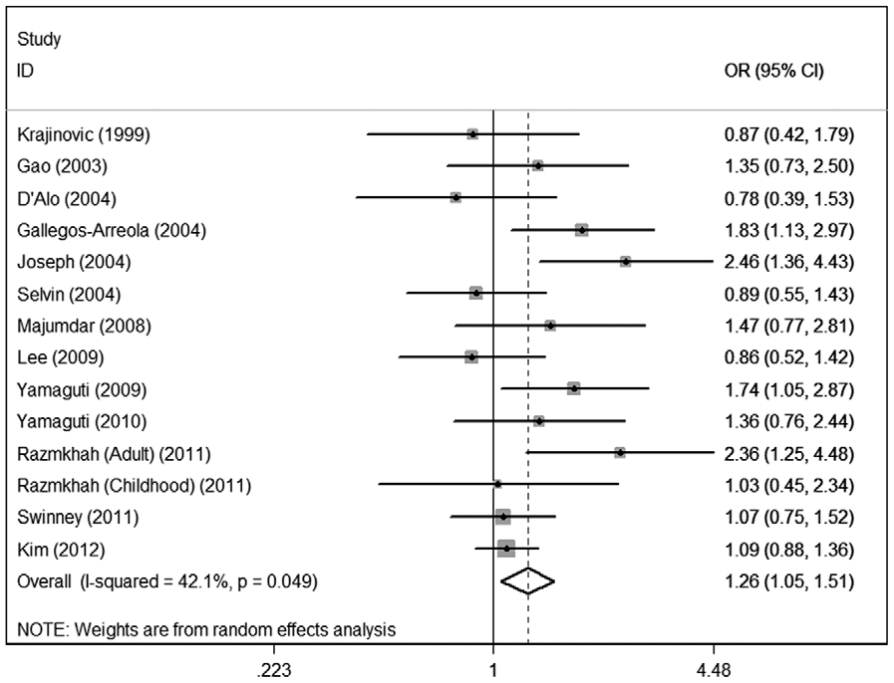
Meta-analysis for the association of acute leukemia risk with CYP1A1 Ile462Val polymorphism for the overall data (Val/Val+Val/Ile versus Ile/Ile).

The main results of the meta-analysis were listed in **Table 3**. For the overall data containing 2164 cases and 4160 controls, significant associations of CYP1A1 Ile462Val polymorphism with acute leukemia risk were shown under the homozygote comparison (OR = 1.49; 95%CI = 1.11–1.98), the dominant (OR = 1.26; 95%CI = 1.05–1.51) **(** **Figure 2** **)** and the recessive (OR = 1.38; 95%CI = 1.04–1.83; P = 0.247 for heterogeneity) models, indicating that individuals carrying the variant Val allele may have an increased acute leukemia risk compared with those bearing the wild-type Ile allele.

**Figure 3:**
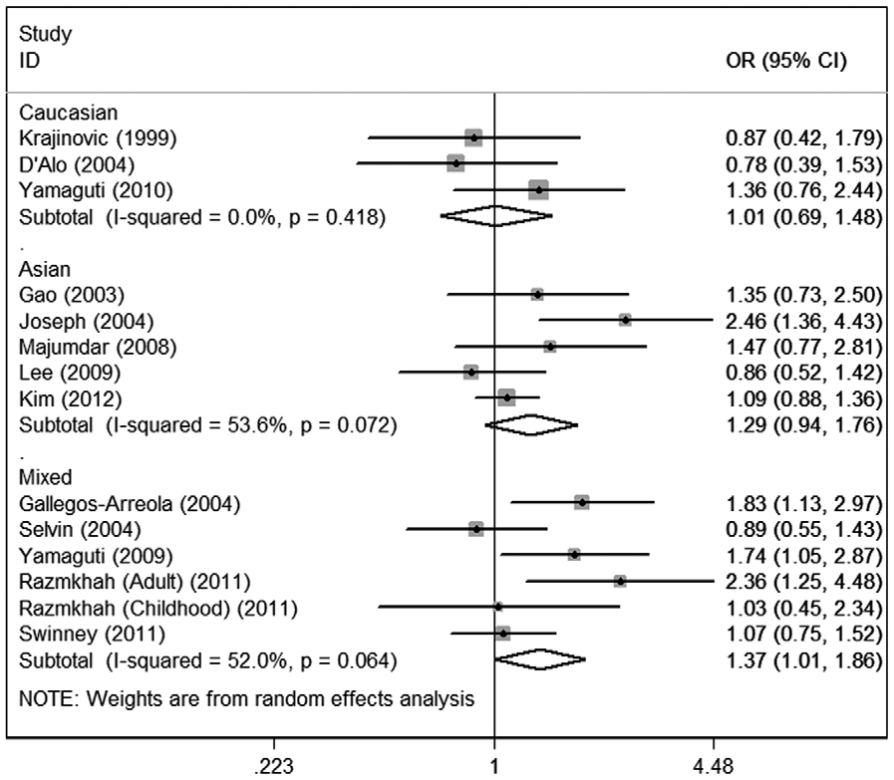
Meta-analysis for the association of acute leukemia risk with CYP1A1 Ile462Val polymorphism (Val/Val+Val/Ile versus Ile/Ile; stratified by ethnicity).

**Figure 4:**
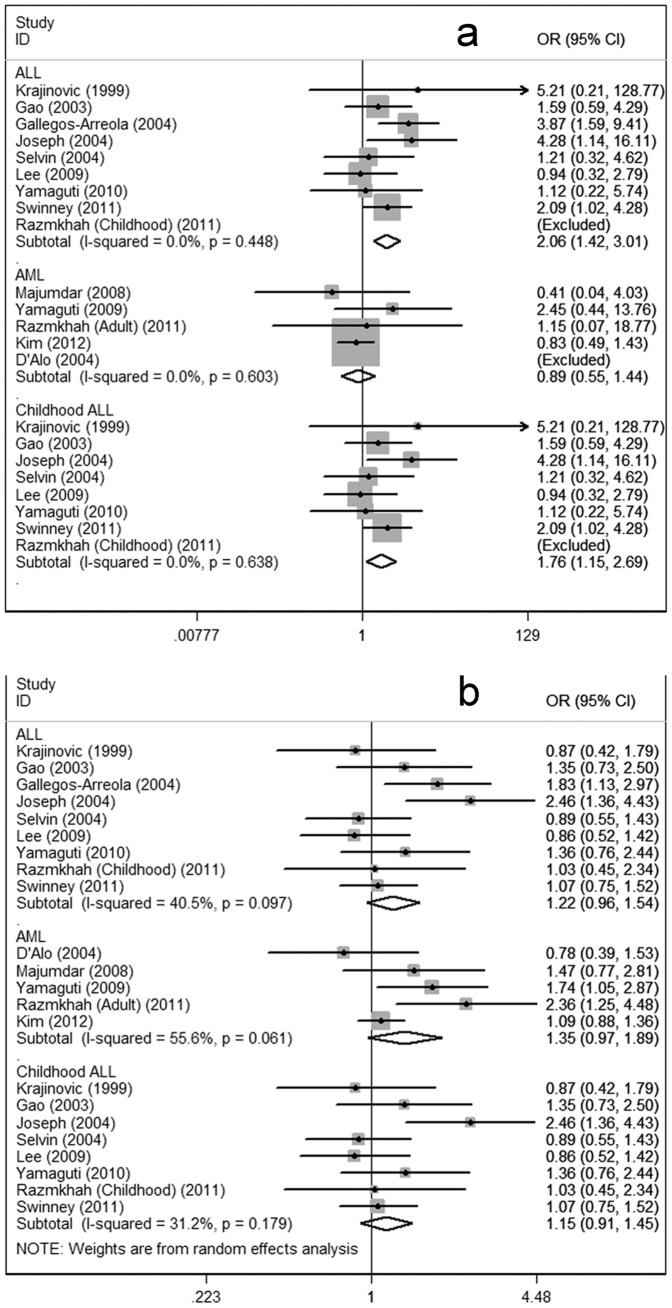
Meta-analysis for the association of acute leukemia risk with CYP1A1 Ile462Val polymorphism stratified by clinical types. (a) Val/Val versus Ile/Ile; (b) Val/Val+Val/Ile versus Ile/Ile; AML, acute myeloid leukemia; ALL, acute lymphocytic leukemia.

**Figure 5:**
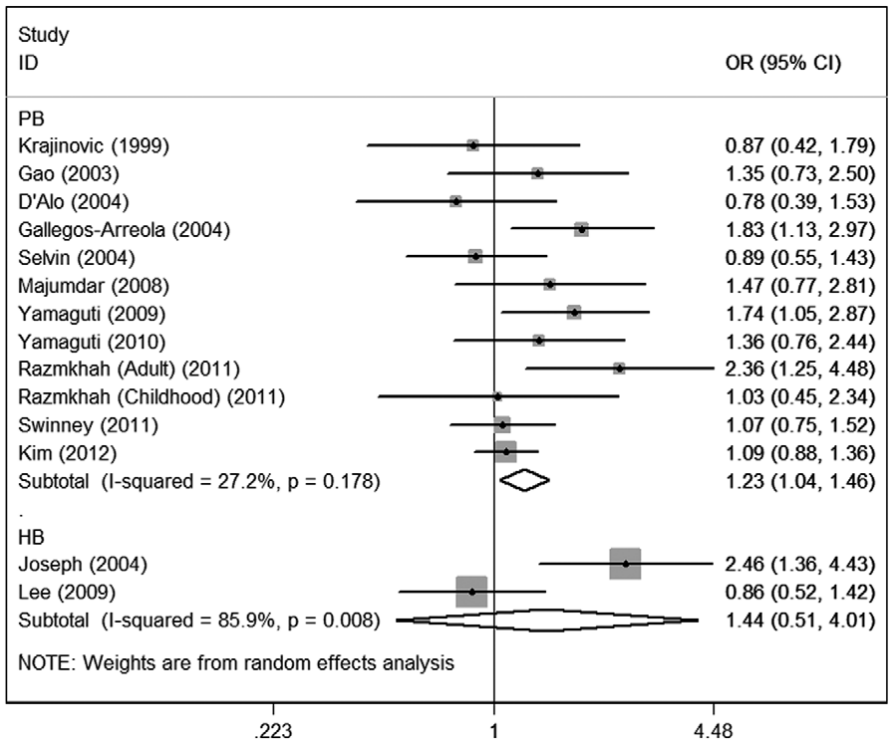
Meta-analysis for the association of acute leukemia risk with CYP1A1 Ile462Val polymorphism stratified by source of controls (Val/Val+Val/Ile versus Ile/Ile). PB: population-based; HB: hospital-based.

Considering the potential impact of the confounding factors on the overall results, we further conducted subgroup analyses. In the primary literature, only the detailed information on ethnicity, clinical types and source of controls were sufficient for analysis. Therefore, subgroup analyses on these issues were performed. In subgroup analysis according to ethnicity, increased leukemia risks were observed in the three genetic models among mixed ethnicities (homozygote comparison: OR = 2.36; 95%CI = 1.46–3.82; dominant: OR = 1.37; 95%CI = 1.01–1.86; recessive: OR = 2.20; 95%CI = 1.37–3.53) but not Caucasians or Asians **(** **Figure 3** **)**. In subgroup analyses regarding clinical types, increased risks for ALL were found under the homozygote comparison (OR = 2.06; 95%CI = 1.42–3.01) and the recessive models (OR = 1.91; 95%CI = 1.32–2.76), respectively. When the data regarding ALL were separated by age groups, significant increased risk could be observed in both adult ALL and childhood ALL. No significant associations were shown in subgroup regarding AML under the three genetic models **(** **Figure 4** **)**. In subgroup analysis on source of controls, elevated risks were observed in the population-based subgroup (homozygote comparison: OR = 1.44; 95%CI = 1.05–1.96; dominant: OR = 1.23; 95%CI = 1.04–1.46) rather than the hospital-based subgroup **(** **Figure 5** **)**.

### 4 Sensitivity Analysis

When the effect-models were changed, the significance of the overall data for the three models was not statistically altered (data not shown). Then, we discarded one study whose genetic distributions in controls exhibited significant deviation from HWE [**35**], given that the deviation might lead to any bias [**37**]. The significances of the overall data in the three models, respectively, were also not statistically changed. Then, one-way sensitivity analysis [**38**] was carried out to assess the stability of the meta-analysis. The statistical significance of the results was not changed when any single study was deleted (data not shown), indicating the credibility of the results.

### 5 Bias Diagnostics

**Figure 6:**
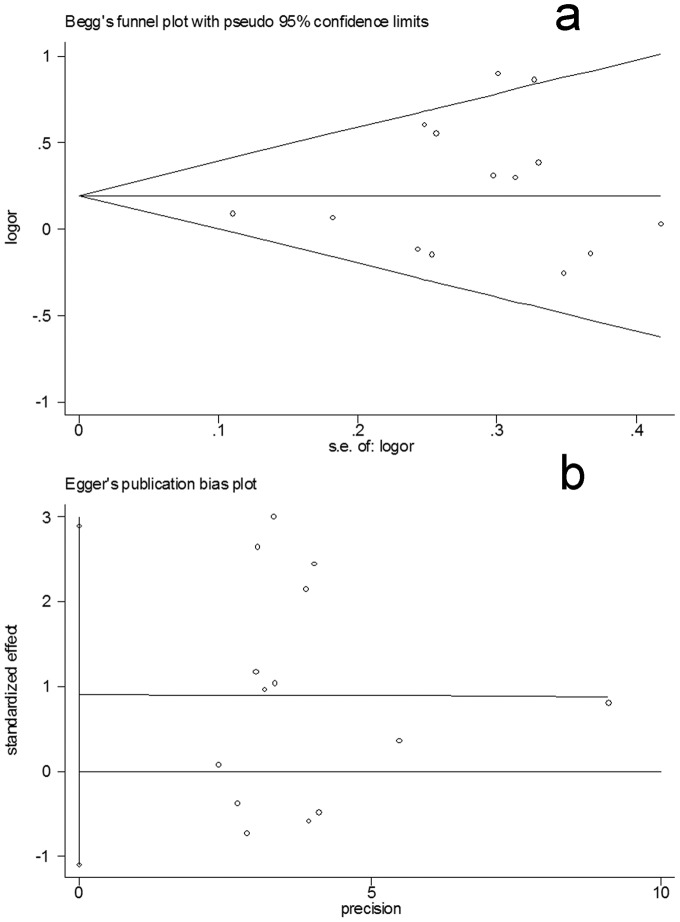
Publication bias tests for the overall data (Val/Val+Val/Ile versus Ile/Ile). (a): Funnel plot; (b) Egger’s linear regression test.

Funnel plots were created to assess the publication bias. Then, Egger’s linear regression tests were used to assess the symmetries of the plots. The funnel plots seemed symmetrical for the overall data of the three genetic models **(** **Figure 6a** **)**. Additionally, the data of the Egger’s tests also indicate the absence of the publication bias in the three models, respectively (homozygote comparison model: t = 0.68, P>0.05; dominant model: t = 0.98, P>0.05; recessive model: t = 0.58, P>0.05) **(** **Figure 6b** **)**, suggesting that the results of the meta-analyses are relatively stable and the potential publication bias might not have an evident influence on the results.

## Discussion

For the overall data, the results showed that CYP1A1 Ile462Val might have a marked correlation with increased acute leukemia risk. Moreover, in subgroup analyses stratified by ethnicity, the data failed to reveal an association among either Asians or Caucasians, but mixed ethnicities. In subgroup analysis according to clinical types, the results indicated that Val/Val alleles might increase susceptibility to ALL.

The relations of CYP1A1 Ile462Val variations with cancer risk have been evaluated by several meta-analyses. CYP1A1 Ile462Val polymorphism might have a correlation with increased risks of lung cancer, cervical cancer, colorectal cancer, esophageal cancer and breast cancers [**39**], [**40**], [**41**], [**42**], [**43**]. Nevertheless, for gastric cancer, prostate cancer and laryngeal cancers [**44**], [**45**], [**46**], such associations were not significant. Thus, CYP1A1 Ile462Val polymorphism might have different influences on different types of malignancies. Recently, a meta-analysis regarding the association of CYP1A1 MspI variation with childhood acute leukemia failed to reveal a significant association [**47**]. However, this meta-analysis focused on CYP1A1 MspI variation but not exon7 (Ile462Val) polymorphism. To our knowledge, the present meta-analysis for the first time shed light on the association between CYP1A1 Ile462Val polymorphism and acute leukemia risk.

In the subgroup analysis according to ethnicity, significant increased leukemia risk was found in the mixed races subgroup but not Asian and Caucasian subgroups, suggesting that CYP1A1 Ile462Val genetic variation may not confer acute leukemia risk among either Asians or Caucasians. Nevertheless, since we could not obtain the data regarding separate ethnicities from the mixed ethnicities subgroup, the possible effects of ethnicity variations on the results could not be precisely evaluated. Evidence suggested that gene polymorphisms could result in ethnic-specific susceptibility to leukemia [**35**]. In addition, environmental factors such as birth place and socioeconomic status may also play critical roles in the genesis of leukemia [**48**]. Therefore, possible racial disparities might exist. Further investigations regarding different ethnicities are needed to clarify this issue.

In the subgroup analysis stratified by clinical types, increased risk for ALL was shown under the homozygote comparison and the recessive models, suggesting that the homozygous Val/Val allele carriers might have an increased ALL risk relative to those of the wild-type Ile carriers. When the data about ALL were further divided by age groups, similar results were obtained in the two subgroups. However, only one study provided the information about adult ALL; thus, the results should be interpreted with care. As for AML, no associations could be observed. The disparity may be due to the different precise mechanisms involved in the genesis of different types of leukemia. ALL is a common childhood leukemia with poor outcomes [**49**], characterized by cytogenetic abnormalities, such as translocations and changes in ploidy. Parental exposure to specific chemicals and widely spread carcinogens may increase risk of childhood ALL [**50**], [**51**]. Hence, variations of CYP1A1 Ile462Val may result in altered activities of the enzymes and the effects of carcinogens on the blood could thus be strengthened. Consequently, ALL risk might be increased.

Smoking and alcohol consumption are also important risk factors for leukemia. Evidence indicates that maternal smoking and drinking prior to and during pregnancy may increase risk of childhood leukemia, particularly ALL [**52**]. We tried to extract relevant information regarding smoking and drinking from the primary literature. However, insufficient data were obtained. Hence, relevant subgroup analyses have not been performed and further investigations concerning the interactions of smoking, drinking and gene variations on leukemia are required.

In the subgroup analysis according to source of controls, significance increased leukemia risk were observed in the population-based subgroup but not the hospital-based group. Since hospital-based controls might not be always truly representative of the general population, any biases might exist and the results should be interpreted with care. Therefore, use of proper control participants with strict matching criteria and large sample sizes are important for reducing such selection bias in future investigations.

In the present meta-analysis, both Q-tests and I-squared values were used to assess the heterogeneities. Evident between-study heterogeneities for overall data were observed in the dominant genetic model, and thus the random-effect models were utilized in this model. However, as shown in **Table 3**, in the subgroup analyses, removed or reduced heterogeneities were found in the relevant subgroups, implying that the heterogeneities may result from multi-factors; in addition to ethnicity and clinical types of leukemia, other factors such as selection of controls, gender, and prevalence of lifestyle factors might also lead to the heterogeneities.

Publication bias is an important factor that should be considered in a meta-analysis. We used funnel plots to evaluate the potential publication bias. Then, Egger’s linear regression test was also used as an approach for assessment of the symmetries. The results failed to suggest evident bias in the three genetic models, suggesting little influences of the bias on the results and demonstrating the robustness and credibility of the present meta-analysis.

Several limitations should be addressed. First, in this meta-analysis, the primary articles only provided data about Caucasians, Asians and mixed ethnicities. Separate data regarding other ethnicities such as African should be concerned. Second, only studies written in English and Chinese were searched and included in this meta-analysis. Thus, any selection bias should be noted. Third, subgroup analyses regarding age, gender and other factors such as smoking, drinking and radiation exposure have not been conducted in the present study because relevant sufficient data were not available in the primary articles. Furthermore, among the fourteen included studies, only three studies provided the adjusted ORs [**31]**, [**32**], [**35**]. We did not pool the adjusted ORs because the included studies either did not adjust for confounders, or the adjustments were not comparable among them. As the adjusted ORs are much more accurate than crude ORs but not available for most included studies, and adjusted factors differed across these studies, residual confounding might have affected the analysis. Therefore, the results should be interpreted with caution. Additionally, gene-gene and gene-environment interactions should also be considered in the further investigations.

In summary, the results of the present meta-analysis suggest that variant Val allele of CYP1A1 Ile462Val polymorphism might have an association with excess acute leukemia risk. Moreover, subgroup analyses indicate that homozygous Val/Val might modify the susceptibility to ALL. Further well-designed investigations in view of the confounding factors are needed reach a more convincible conclusion.
